# STAT3 activation in monocytes accelerates liver cancer progression

**DOI:** 10.1186/1471-2407-11-506

**Published:** 2011-12-05

**Authors:** Wen-Yong Wu, Jun Li, Zheng-Sheng Wu, Chang-Le Zhang, Xiang-Ling Meng

**Affiliations:** 1 Department of General Surgery, First Affiliated Hospital of Anhui Medical University, Anhui, Hefei, People's Republic of China; 2 School of Pharmacy, Anhui Medical University, 81 Meishan Road, Hefei, Anhui 230032, People's Republic of China; 3 Department of Pathology, Anhui Medical University, Hefei, Anhui, People's Republic of China

**Keywords:** STAT3, Liver cancer, Inflammation

## Abstract

**Background:**

Signal transducer and activator of transcription 3 (STAT3) is an important transcription factor ubiquitously expressed in different cell types. STAT3 plays an essential role in cell survival, proliferation, and differentiation. Aberrantly hyper-activated STAT3 signaling in cancer cells and in the tumor microenvironment has been detected in a wide variety of human cancers and is considered an important factor for cancer initiation, development, and progression. However, the role of STAT3 activation in monocytes in the development of HCC has not been well understood.

**Methods:**

Immunohistochemical analysis of phosphorylated STAT3 was performed on tissue microarray from HCC patients. Using a co-culture system in vivo, HCC cell growth was determined by the MTT assay. In vivo experiments were conducted with mice given diethylinitrosamine (DEN), which induces HCC was used to investigate the role of STAT3 expression in monocytes on tumor growth. Real-time PCR was used to determine the expression of cell proliferation and cell arrest associated genes in the tumor and nontumor tissue from liver.

**Results:**

Phosphorylated STAT3 was found in human hepatocellular carcinoma tissue samples and was expressed in tumor cells and also in monocytes. Phosphorylated STAT3 expression in monocyte was significantly correlated to advanced clinical stage of HCC and a poor prognosis. Using a co-culture system in vivo, monocytes promoted HCC cell growth via the IL-6/STAT3 signaling pathway. The STAT3 inhibitor, NSC 74859, significantly suppressed tumor growth in vivo in mice with diethylinitrosamine (DEN)-induced HCC. In this animal model, blockade of STAT3 with NSC 74859 induced tumor cell apoptosis, while inhibiting both tumor cells and monocytes proliferation. Furthermore, NSC 74859 treatment suppressed cancer associated inflammation in DEN-induce HCC.

**Conclusion:**

Our data suggest constitutively activated STAT3 monocytes promote liver tumorigenesis in clinical patients and animal experiments. Thus, STAT3 in tumor infiltrating inflammatory cells may an attractive target for liver cancer therapy.

## Background

A causal link between chronic inflammation and the development of cancer has long been recognized from clinical and animal investigations and has become an issue of high interest in recent years [1]. Although it is well accepted that chronic inflammation can affect all phases of carcinogenesis, from the initial cancer formation by inducing genetic alteration, to the subsequent cancer formation by establishing an inflammatory environment that allows tumors to grow, metastasize and escape the host anti-tumor immune response [1,2], the exact mechanisms of inflammation favoring carcinogenesis are largely currently unknown. The interplay between chronic inflammation and cancer is very complex. Previous reviews have already demonstrate this interesting issue in detail [1,2]. Briefly, chronic inflammation and incomplete repair can hasten the oncogenic processes by directly promoting genetic instability and favoring the induction of gene mutation. It is believed abundant reactive oxygen (ROS) produced by inflammatory cells can induce DNA damage, mutations and genetic instability. Secondly, many well known oncogenic genes including RAS, RET, BRAF and MYC appear to play a role in inflammation as well [3]. These oncogenes turn on the inflammatory pathway within a cell, activate inflammation outside the cell to recruit inflammatory cells leading to an immuno-suppressive tumor microenvironment [2,4,5]. Lastly, many transcription factors such as NF-κB, STAT3 and the adaptor protein MyD88, which are all key to the innate inflammatory response, are also essential in certain kinds of cancers [6-9].

Constitutively activated IL-6/STAT3 signaling has been detected in a wide variety of human cancers including liver cancer and is considered an important factor for cancer initiation, development, and progression [7,10-12]. Hepatocellular carcinoma (HCC) is the most common primary malignancy in liver and the third leading cause of cancer deaths worldwide, with few effective therapeutic options for this severe disease [13-15]. Most HCC appears in cirrhotic livers after years of chronic liver inflammation caused by hepatitis viral infection, alcoholic and non-alcoholic steatohepatitis [14,16]. Various factors can active hepatic STAT3 signaling such as inflammatory cytokines, growth factors, hormones, and hepatitis viral proteins [17]. Several cytokines (such as IL-6, IL-6 family cytokines and IL-22) that activate STAT3 in hepatocytes have been shown to promote HCC cell growth in vivo and in vivo [18,19]. Recently, Park et al. reported that localized production of IL-22 in the liver promotes hepatocyte survival and proliferation, thereby accelerating the HCC development after DEN challenge [20]. Moreover, emerging evidence suggests that the cytokines downstream of STAT3 play an important role in the development of liver cancer [21-23]. Blockage of STAT3 may have therapeutic potential in preventing and treating liver cancer [24-26]. Our previous study on HCC specimens suggests an oncogenic role of STAT3 in liver cancer. In the previous study tumor expression of STAT3 was correlated with disease progression and poor survival rates [27]. In the present study we further investigate whether monocyte expression of STAT3 in the tumor microenvironment could promote tumor growth and whether the STAT3 inhibitor, NSC 74859, can prevent diethylinitrosamine (DEN)-induced HCC by suppressing STAT3 activation and its associated inflammation.

## Methods

### Cancer specimens

A total of 138 HCC patients were enrolled in this study with an informed patient consent following the human study protocol approved by the Anhui Medical University Ethics Committee. Formalin-fixed and paraffin-embedded HCC and normal liver specimens were obtained from the Department of Pathology within the First Affiliated Hospital of Anhui Medical University, P.R. China. All HCC samples were collected from patients with varying grades and stages of cancer. Two independent pathologists evaluated blinded tumors samples used in this study. All the hematoxylin and eosin-stained sections from each paraffin-embedded, formalin-fixed block were reviewed to identify target areas.

### Tissue microarrays (TMA) construction

Paraffin-embedded tumor specimens were obtained from an archive of the Department of Pathology within the First Affiliated Hospital of Anhui Medical University, P.R. China. TMAs were constructed as previously described [27]. Three to five representative 1 mm cores were obtained from each case and inserted in a grid pattern into a recipient paraffin block (Hengtai Instruments Inc., Liaoning, P.R. China).

### Immunohistochemistry staining

TMA sections of HCC were stained through immunohistochemistry using primary antibodies against ^pY705^STAT3 (Cell Signaling Technology, Danvers, MA, USA). The frequency of p^Y705^STAT3-positive cells was measured by counting the total number of cells and the number of positively stained cells. More than 25% nuclear staining was classified as positive.

For cell proliferation Ki67 staining, the sections were stained in accordance with routine immunohistochemistry procedures and visulaized with the ABC kit (Vector Laboratories, Burlingame, CA, USA). Biotinylated rat anti-mice Ki67 antibody at 1:100 dilution was used. Hepatocyte or tumor cell apoptosis was detected by using an Apoptag Apoptosis Detection Kit (Chemicon International, Temecula, CA, USA).

### Co-culture of monocytes and HCC cells

The HCC cell lines HepG2 and Huh-7 were obtained from the Shanghai cell bank, Chinese Academy of Sciences, Shanghai, China. These two cell lines were maintained in Dulbecco's Modified Eagle's Medium (DMEM). Both types of medium were supplemented with 10% fetal bovine serum.

PBMCs from health subjects were freshly isolated by gradient centrifugation from discarded leukocyte filters obtained during platelet collection from healthy adults at the First Affiliated Hospital of Anhui Medical University. Monocytes were isolated by negative selection using Dynal Monocyte Negative Isolation Kit (Invitrogen) according to the manufacturer's instructions. All in vivo experiments were performed in Ultra Low Attachment Plates (Corning) to prevent monocyte activation by adhesion to the plastic plate. In co-culture experiments, freshly isolated monocytes (5 × 10^6^) were added to the inserts separated by 0.4-μm membrane (Costar; Corning) from HCC cells. For the cell proliferation analysis, HCC cell line HepG2 or Huh-7 were co-cultured with or without moncytes for 48 h and the MTT assay was performed as described below.

### 3-(4, 5-Dimethylthiazol-2-yl)-2, 5-diphenyltetrazolium bromide (MTT) assay

The MTT assay is based on the conversion of the yellow tetrazolium salt MTT to purple formazan crystals by metabolically active cells. The MTT assay provides a quantitative determination of viable cells. Cells (1 × 10^4^) were seeded in 96-well microplates in complete culture medium in the absence or presence of anti-IL-6 antibody or NSC 74859 as indicated. After 72 h of culturing, the number of viable cells was measured by adding 100 μl/well of 2 mg/ml MTT solution. The medium was removed 2 h later and the formazan crystals were dissolved by adding 100 μl dimethylsulfoxide per well. The absorbance was read at 590 nm with an enzyme-linked immunosorbent assay reader. Each treatment point was performed with an n = 6.

### Mice and diethylnitrosamine (DEN)-induced liver cancer model

All experiments with mice were approved by Anhui Medical University Animal Care and Use Committee. C57BL/6 mice were obtained from Animal Center of Anhui Medical University. Mice were kept in pathogen-free conditions with room temperature of (23 ± 2C), humidity (55-60%), and light conditions (12 h light/dark cycle).

The DEN-induced liver tumor model in mice was established as described previously [28,29]. Briefly, 15-day-old B6 mice were injected with 5 μg/g DEN (Sigma, St. Louis, MO). Six months after the injection series with normal chow, all the mice were randomly separated into two groups; the NSC 74859 group or vehicle only group. Mice were then injected intraperitoneally with 5 mg/kg NSC 74859 twice per week for 3 months prior to sacrifice at 9 months after DEN injection. Livers were removed and the tumor numbers and sizes were analyzed. Histological sections were taken including larger tumor nodules that were fixed in 10% formalin. Hematoxylin-eosin (H&E) staining was performed using standard protocols. The liver tumor tissues and nontumor tissues were carefully separated and frozen in liquid nitrogen for subsequently real-time PCR determination.

For liver tumor analysis, the whole liver was carefully removed from the euthanized animal, washed and placed in cold PBS. The numbers of surface liver tumor nodules were counted for all liver lobes in a blinded fashion. Liver nodules typically presented as basophilic foci with crowded nuclei and were classified as atypical foci (HCC) or hepatocellular adenomas.

### Real-time PCR

Real-time PCR was used to determine the expression of cell proliferation and cell arrest associated genes in the tumor and nontumor tissues from liver samples. Total RNA was purified from approximately 30 mg of liver tumor or nontumor samples according to the manufacturer instruction (Qiagen). 1 μg of mRNA was reverse-transcribed to cDNA using a High Capacity cDNA Reverse Transcription kit (Invitrogen). cDNA templates were diluted 1:5 and amplified using real-time PCR through the iTaq SYBR Green Supermix (Bio-Rad, Hercules CA). An initial denaturation at 95°C for 3 min was followed with PCR cycling: 95°C (15 sec), and 58°C (30 sec) for 40 cycles. Relative mRNA levels were calculated by means of 2^- ΔΔ Ct ^(ΔΔ Ct = difference of crossing points of test samples and respective control samples as extracted from amplification curves by the LightCycler software) after normalization to 18S rRNA expression, which was used as an internal standard. Fold inductions of analyzed mRNA expression were normalized on 18S rRNA expression. PCR was performed with 12.5 μl SYBR Master Mixture and the following primers in Table 1.

**Table 1:** Primer sequences for real-time PCR

genes	Forward primer (5'...3')	Reverse primer (5'...3')
TNF-α	AAGCCTGTAGCCCACGTCGTA	AGGTACAACCCATCGGCTGG

IL-1β	AAAAAAGCCTCGTGCTGTCG	GTCGTTGCTTGGTTCTCCTTG

IL-6	TCCATCCAGTTGCCTTCTTG	TTCCACGATTTCCCAGAGAAC

MCP-1	TCAGCCAGATGCAGTTAACGC	TCTGGACCCATTCCTTCTTGG

IFN-γ	GCCCTCTCTGGCTGTTACTG	CTGATGGCCTGGTTGTCTTT

18 s	GTAACCCGTTGAACCCCATT	CCATCCAATCGGTAGTAGCG

### Statistical analysis

All statistical analyses were performed using SPSS software system for Windows (version 13.0; SPSS, Chicago, IL, USA). Differences between groups were compared using Pearson's chi-square test for qualitative variables and Student's *t*-test for continuous variables. Kaplan-Meier curves were constructed to determine patient relapse-free survival (RFS) and overall survival (OS) rates. The statistical differences in survival among subgroups were compared using the log-rank test. Data of HCC mice model were expressed as means ± SE (N = 4-8 in each group). To compare values obtained from three or more groups, 1-factor analysis of variance (ANOVA) was used, followed by Tukey's post hoc test. *P *< 0.05 was considered statistically significant. The correlations between variables were assessed by the Spearman rank order test. Statistical significance was taken at the *P *< 0.05 level.

## Results

### Phosphorylated STAT3 in hepatocellular carcinoma tissue is not only expressed on tumor cells, but also on monocytes

**Figure 1:**
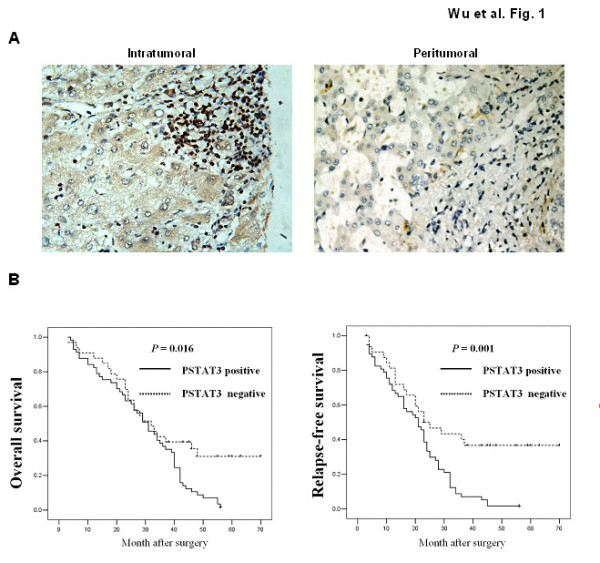
**STAT3 activation in monocytes in HCC patients**. (**a**) Representative images of pSTAT3 immunostaining in intratumoral (left) and adjacent peritumoral (right) tissues are shown. (**b**) Overall survival and relapse-free survival for HCC patients according to presence of pSTAT3 expression in monocytes.

Previous study have shown that STAT3 and pSTAT3 expression is dramatically increased in HCC tissues when compared with normal liver samples [22]. To further investigate which cell types, in addition to tumor cells, were responsible for pSTAT3 activation and the STAT3 signaling and whether this signaling pathway was correlated with the prognosis of HCC patients, we initially used immunohistochemistry to analyze pSTAT3 expression in HCC tissues and its association with patient's clinicopathological parameters (Table 2). Positive pSTAT3 staining in monocytes and overall survival was evaluated using Kaplan-Meier survival curves and the log-rank test (Figure 1b). After reviewing the TMA slides of HCC, we found 113 out of 138 HCC cases had the inflammation cells infiltration. Among the 113 patients studied, 92 patients were classified as HBsAg positive and 101 patients were classified as cirrhosis patients. In the non-peritumoral area in tumors from these patients, the inflammatory cell infiltration was generally dense, as seen in the cirrhotic stroma, and several pSTAT3 positive monocytes were detected (not taken into account in the evaluation). To clarify the interaction between the monocytes and tumor cells in the tumor microenvironment, pSTAT3 expression was investigated in the limited areas of the peritumoral and the intratumoral area. As shown in Table 3, a statistically significant difference of pSTAT3 was observed in tumor cells (54.3%) and monocytes (60.2%) when compared with adjacent non-tumorous tissue. As shown in Table 4, a statistically significant correlation was seen between pSTAT3 expression in tumor cells and its adjacent pSTAT3 positive monocytes by Spearman correlation analysis(*P *< 0.001, rs = 0.440). For the non-tumoral liver tissue (tissue away from the peritumoral and intratumoral area), the pSTAT3 expression was weak or scarce except for the inflammatory foci area. These data are summarized in Tables 2, 3 and 4. Interestingly, expression of pSTAT3 in monocytes was increased in intratumoral tissues compred to adjacent peritumoral tissues (Figure 1a). Monocyte expression of pSTAT3 was significantly correlated to higher clinical stages of HCC (*P *= 0.032, Table 1). Next, we further identify the correlation of pSTAT3 expression in monocytes with the prognosis of HCC inpatients. As shown in Figure 1b, HCC patients with enhanced pSTAT3 expression in monocytes had a significantly worse OS and RFS after curative resection than those without pSTAT3 expression (*P *= 0.016 and 0.001, respectively).

**Table 2:** Correlation of pSTAT3 expression in moncytes with clinicopathological parameters from HCC patients (n = 113)

Parameter	*n*	pSTAT3 positive expression, *n *(%)	*p *value
Age (years)			

≤ 55	67	45 (67.2)	0.067

> 55	46	23 (50.0)	

Gender			

Male	93	55 (59.1)	0.627

Female	20	13 (65.0)	

Cirrhosis			

Yes	101	63 (62.4)	0.166

No	12	5 (41.7)	

HBsAg			

Yes	92	58 (63.0)	0.193

No	21	10 (47.6)	

Tumor size (cm)			

< 5	32	17 (53.1)	0.336

≥ 5	81	51 (63.0)	

Grade			

I	4	2 (50.0)	0.472

II	107	64 (59.8)	

III	2	2 (100.0)	

Stage			

I-II	86	47 (54.7)	0.032

III-IV	27	21 (77.8)	

Ki67			

< 50%	45	24 (53.3)	0.227

≥ 50%	68	44 (64.7)	

**Table 3:** Expression of pSTAT3 in HCC and adjacent non-tumour tissue specimens

Group	*n*	Positive expression of pSTAT3 protein, *n *		
		
		tumor/liver cells	*n*	monocytes
HCC	138	75 (54.3)*	113	68 (60.2)*

Non-tumorous	110	35 (31.8)	110	27 (24.5)

*P < 0.001

**Table 4:** Correlation analysis of pSTAT3 expression between in tumor cell and monocyte in HCC

Monocyte expression of pSTAT3	Tumor cell expression of pSTAT3	
	
	Negative	Positive
Negative	87 (68.0)	41 (32.0)

Positive	38 (40.0)	57 (60.0)

*P < 0.001, r_s _= 0.440

### Monocytes promote HCC cells growth via IL-6/STAT3 pathway

**Figure 2:**
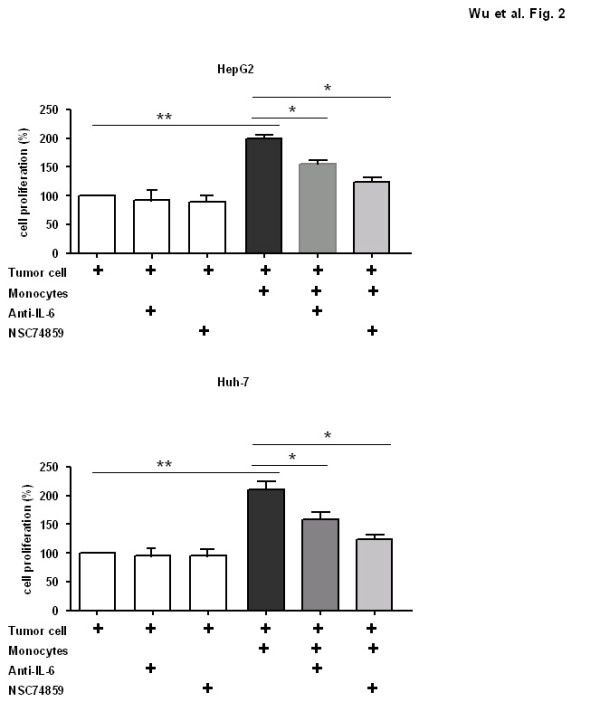
**Monocytes promoted HCC cell proliferation in vitro via IL-6/STAT3 signaling pathway**.

To further analyze the underlying mechanism of the poor prognosis in HCC with increased expression of monocyte STAT3, we cultured HCC cell lines HepG2 or Huh-7 alone, or co-cultured these cells using transwell chambers with HCC cells in the presence of peripheral blood-derived monocytes. As shown in Figure 2, cell proliferation of HepG2 or Huh-7 was much higher in co-cultures of both cell types for 24 hrs when compared with single culture of HCC cells. Pretreated with an IL-6 antibody or STAT3 inhibitor significantly suppressed HCC cells growth. Cell proliferation was comparable between HCC cells only and tumor cells treated with or without IL-6 antibody or STAT3 inhibitor. These in vivo data clearly show that activated STAT3 in monocytes can promote cancer cells growth in a paracrine dependent manner.

### Inhibition of STAT3 with NSC 74859 results in tumor regression in DEN-induced HCC mouse model

**Figure 3:**
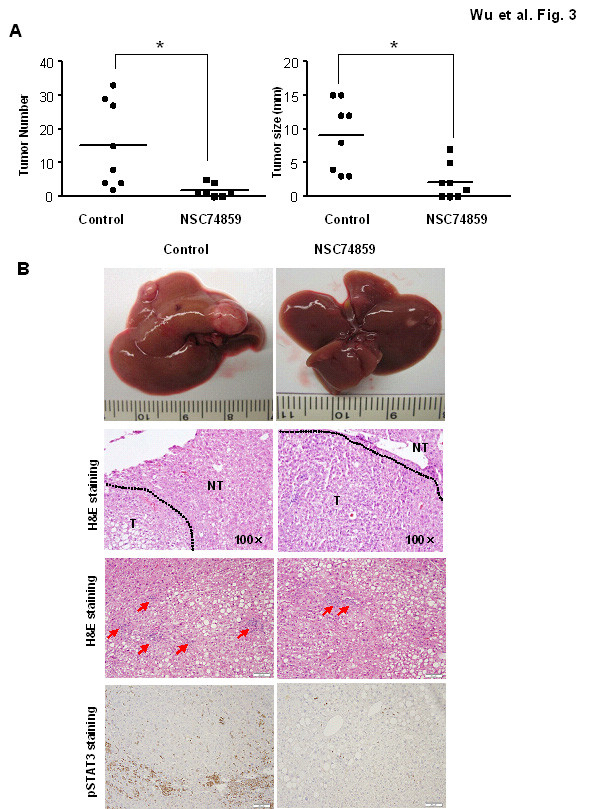
**NSC 74859 reduced DEN-induced liver tumorigenesis (**a**) The mice from control and NSC 74859 group treated with 5 μg/g of DEN at age 15 days and sacrificed post-DEN (8 mice per group)**. The number and size of tumors on surface were counted. (**b**) Representative images of livers, H&E staining, inflammation and pSTAT3 immunostaining are shown. T: tumor; NT: non-tumor tissues. The arrows in inflammation analysis indicate the inflammatory foci.

Previous studies have shown that STAT3 inhibitors can suppress tumor cell growth in vivo and tumor growth in a tumor xenograft mouse model [22]. Here, using the traditional DEN-induced HCC model, we investigated the effects of the STAT3 inhibitor, NSC 74859, on HCC development. Both the control and the STAT3 inhibitor treated mice (8 mice per group) were injected with DEN at day 15. Both NSC 74859 and the vehicle alone were injected intraperitoneally at 5 mg/kg twice per week for 3 months prior to sacrificing the mice at 9 months after DEN injection. The numbers of surface liver tumor nodules from all liver lobes were enumerated and the sizes of the tumor nodules were also calculated. As illustrated in Figure 3, injection of NSC 74859 inhibited tumor development in the DEN-induced tumor model. The tumor size and number were significantly smaller in NSC 74859 treated mice compared to wild-type mice after injection of 20 μg/g of DEN.

Histological analysis (Figure 3) shows that liver tumors from both groups had a moderate degree of differentiation with an increased nuclear-to-cytoplasmic index, enlarged and hyperchromatic nuclei and expansive growth. In the area of the tumor tissues, the normal liver architecuture, such as bile duct and portal tract formation, was lost. Consistent with the results in Figure 3a, the tumor size was much smaller after NSC 74859 treatment. These data clearly demonstrate the anti-tumor capacity of NSC 74859 in DEN-induce HCC development. Immunohistochemical staining analysis also shows stronger pSTAT3 expression in both tumor and inflammatory cells compared to the vehicle control treatment mice, while NSC74859 markedly decreased pSTAT3 expression (Figure 3). Hematoxylin and eosin staining indicate a large inflammatory cell infiltration in DEN-treated mice, compared to vehicle control mice. Inflammation reduced in the adjacent area to tumor tissue after NSC 74859 therapy (Figure 3).

### Blockade of STAT3 with NSC 74859 induced tumor cell apoptosis, while inhibited both tumor cells and monocytes proliferation in DEN-induced HCC mouse model

**Figure 4:**
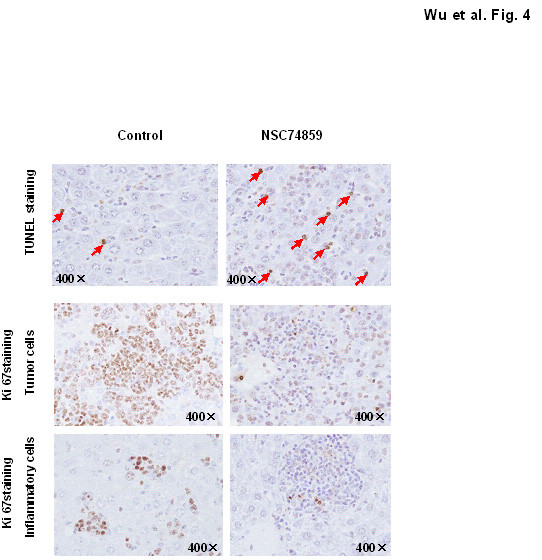
**NSC 74859 promoted tumor cell apoptosis while inhibited tumor cell and monocytes proliferation Representative images of TUNEL staining and Ki67 immunostaining are shown**.

To explore the possible mechanisms underlying the anti-tumor effects of the STAT3 inhibitor in this HCC model, we performed TUNEL and Ki67 staining in liver tumor tissue after DEN exposure in both groups. As shown in Figure 4, administration of NSC 74859 led to more apoptotic tumor cells and less Ki67^+ ^tumor cells. Moreover, NSC 74859 also reduced the cancer-associated inflammatory cell proliferation.

**Figure 5:**
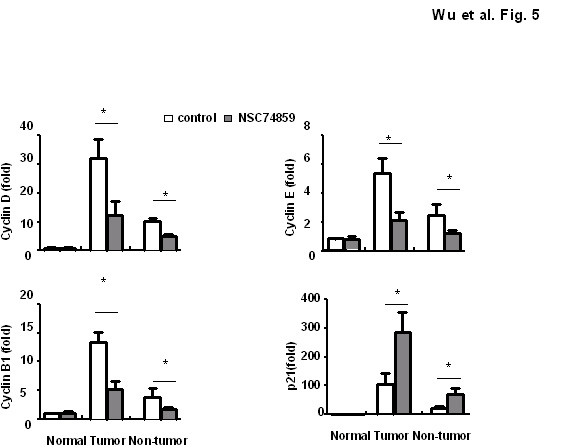
**NSC 74859 regulated cell cyclin gene expression in DEN-induced HCC mice**. mRNA in T and NT tissues were analyzed by real-time PCR (n = 4).

Constitutively activated STAT3 promotes tumorigenesis through the upregulation of cell cyclin related gene [12,30-32]. Therefore, we compared the cell cyclin related gene expression (Cyclin B1, Cyclin D, Cyclin E and p21) between the NSC 74859 treatment and control groups through real-time PCR. The expression of Cyclin B1, Cyclin D and Cyclin E in NSC 74859 treated mice were low compared with the untreated mice in both tumor tissue and non-tumor tissue, as shown in Figure 5, which was correlated with the reduced proliferation in the NSC 74859 treated group. In contrast, the level of p21 was higher in NSC 74859 group compared with the untreated controls.

### NSC 74859 suppressed cancer associated inflammation in DEN-induce HCC

**Figure 6:**
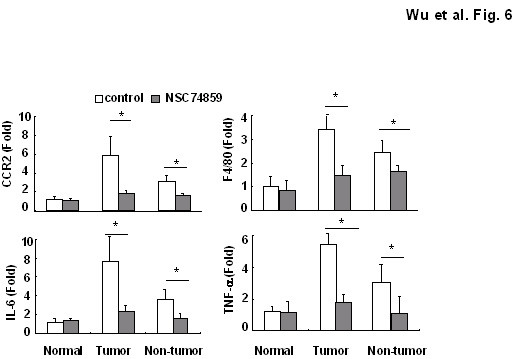
**NSC 74859 ameliorated cancer-associated inflammation in DEN-induced HCC mice**. mRNA in T and NT tissues were analyzed by real-time PCR (n = 4).

Since a causal relationship between chronic injury and inflammation and liver carcinogenesis in the majority of HCC patients is well established, we wondered whether the STAT3 inhibitor can also affect liver inflammation in the DEN-induced HCC model. To test this possibility, the mRNA expression of several pro-inflammatory cytokines (IL-6 and TNF-α) and inflammatory markers of macrophages (F4/80) and monocytes (CCR2) were examined using real-time PCR in mice with or without NSC 74859 treatment. Interestingly, as illustrated in Figure 6, both tumor and non tumor tissue after STAT3 inhibitor administration displayed less expression of inflammatory cell marker and cytokines compared with those in untreated tissue. Those data indicated the STAT3 inhibitor also can reduce inflammation after DEN challenge.

## Discussion

Although transcription factors such as NF-κB and STAT3, are key molecules implicated in cancer-related inflammation [21-23,33-36], the current study provides several novel findings demonstrating the importance of monocytes STAT3 activation in facilitating HCC progress in human patients and in an animal model. First, a negative correlation was observed between STAT3 activation in monocytes and overall survival in human HCC patients. Second, in co-culture experiemtns with monocytes and tumor cells, monocytes enhance HCC cell proliferation, which was dependent on IL-6/STAT3 signaling pathway. And finally, STAT3 inhibitor treatment in DEN-induced HCC animal not only reduced tumor growth but also ameliorated cancer associated inflammation via inhibiting inflammatory cell STAT3 activation. These finding indicates that monocyte-dervied STAT3 is a possible new therapeutic target for HCC.

The presence of inflammatory cells including monocytes in the tumor microenvironment has been widely reported. The function of these cancer associated inflammatory cells is complicated and generally viewed as both beneficial as anti-tumorogenic and tumor promoting in regards of the immune response. In the present study, we observed monocytes infiltrating the peritumoral and intratumoral area of HCC and the associated STAT3 activation, which was statistically significant and associated with poor prognosis in these cells is prominent. At present, the underlying mechanisms for these inflammatory cells are not well known. Previous studies indicate that the immunosuppressive response, angiogenic factors and tumor-promoting chemokines induced by infiltrating inflammatory cells contribute to tumor growth and metastasis [10]. Recently, IL-17 and IL-21, synthesized by immune cells has been shown to promote tumor development in inflammation-associated cancers [37,38]

Our results, along with the study in other different types of cancer [7,10-12], indicate the existence of an association between STAT3 activation in monocytes and poor prognosis. (Figure 1). This observation in clinical setting suggests that tumor-infiltrating monocytes STAT3 expression may have a protumoral function. Although most of patients in our study have the history of HBV infection, it is very difficult to clarify the relationship between natural history of the HBV infection and STAT3 activation. HCC is the very late stage of severe liver disease and survival time of the patients is limited. One interesting finding showed that STAT3 expression and phosphorylation was not altered in HCV-fibrosis patients and alcoholic cirrhosis, while STAT3-DNA binding was markedly suppressed in all alcoholic and most HCV fibrosis patients when compared with that in normal healthy livers[39,40]. Elucidating the roles STAT3 in HBV infection and HBV inducing neoplastic transformation will shed light on the molecular basis of liver cancer and may suggest therapeutic strategies for this severe disease.

IL-6 is a multifunctional cytokine which is known to affect proliferation, apoptosis and angiogenesis in cancer [41]. In liver disease, clinical data also indicate that serum IL-6 concentrations are elevated in patients with chronic liver inflammation, and steatohepatitis as well as in patients with HCC [42]. Notably, men are about three to five times more likely to develop HCC than women [43]. Similar gender disparity was also observed in a murine model of HCC induced by diethylnitrosamine (DEN). It is believed that higher serum levels of IL-6 in male mice contribute to the increased susceptibility to DEN-induced liver cancer in these mice compared with female mice [19]. Since IL-6 can strongly activate the STAT3 signaling pathway, it is reasonable to expect STAT3 also plays a critical role in HCC development. Indeed, a previous study has already reported constitutively activated STAT3 in human liver tumor tissues [22]. Consistent with this study, we also found STAT3 activation in tumor cell. Moreover, we observed STAT3 activation in infiltrated monocytes adjacent to tumor tissue (Figure 1a). Activated STAT3 in monocytes are positively correlted with a poor prognosis (Figure 1b). Previous study showed that, strong STAT3 immunostaining was observed in the cytoplasm of HCC tissues, while pY705STAT3 immunostaining was observed in the nucleus [22]. Additionally, blockage of STAT3 using chemical inhibitors or siRNA induced liver cancer cell apoptosis and cell cycle arrest in vivo, and inhibited growth of transplanted liver cancer cells in vivo [22]. In this study, we observed that altered p-STAT3 expression was significantly and positively correlated with the histological grading and intratumor microvessel density in HCC. Interestingly, recent studies suggest that STAT3 activation is also implicated in HCV- and obesity-mediated hepatocarcinogenesis [35,36]. Another important evidence for the role of STAT3 in liver cancer development is that constitutively activated STAT3 is detected in cancer stem cells from HCC and likely contributes to liver cancer stem cell proliferation and survival [44]. Collectively, Activation of STAT3 in cancer cells plays an important role in liver tumorigenesis.

The oncogenic role of constitutively activated STAT3 is driven through the up-regulation of cell survival proteins (Bcl-xl, Bcl-2), cell cycle regulators (c-Myc, cyclin D) [12,30-32], anti-oxidant genes (Mn-SOD, ferritin, catalse), and tissue repair genes (Reg β, Reg γ, Tff3) [31,45,46]. Our study also showed that STAT3 inhibitor treatment down-regulated cell proliferation-related gene expression. Recently, a key novel molecule, sphingosine-1-phosphate receptor-1 (S1PR1) that is induced by STAT3, has been discovered to play an important role in inducing persistent STAT3 activation in tumor cells and in the tumor microenvironment [47].

Besides promoting tumor cell proliferation and inhibiting cell apoptosis [31,48,49], the activation of STAT3 in cancer cell has also shown to increase the capacity of tumor to evade the immune system, by inhibiting the maturation of dendritic cells and suppressing the immune response [7,50]. Overexpression of STAT3 in tumor cells can recruit tumor-infiltrating hematopoietic cells by producing chemotactic factors, resulting in infiltrating inflammatory immune cells and subsequently STAT3 activation in immune cells. The interplay of STAT3 in cancer cells and immune cells in tumor microenvironment is very complex and remains elusive. Previous studies show that the persistent activation of STAT3 in immune/inflammatory cells is also very important in the control of tumor promotion and progression through tumor-promoting inflammation and suppressing anti-tumor immunity[51-53]. Our study demonstrates that in vivo monocytes can promote liver cancer cell proliferation via IL-6/STAT3 signaling pathway (Figure 2). This is direct evidence to demonstrate STAT3 in monocytes can broadly and profoundly affect tumor growth via stimulation of tumor cell survival and proliferation. In vivo, STAT3 inhibitors can also decrease cancer-associated inflammation, suggesting that targeting leukocyte STAT3 in the tumor microenvironment may be a therapeutic option that will be applicable in the future. However, these results are in contrast to a previous report [54]. In this report, the authors indicate that the deletion of STAT3 in myeloid cells, including leukocytes, enhances inflammation in concanavalin A-induced hepatitis. These results suggest that STAT3 inhibition in immune cells leads to enhanced inflammation. These conflicting observations indicate the complexity of molecular mechanisms underlying liver inflammation and cancer. Decreased tumor-associated inflammation induced by STAT3 inhibitor may be a secondary response after the inhibition of STAT3 in tumor cells. Future studies will determine why STAT3 inhibitors decrease tumor-associated inflammation while enhancing necrotic-associated inflammation.

## Conclusions

Our study clearly suggests constitutively activated STAT3 monocytes promoting liver tumorigenesis in clinical patients and animal experiments. STAT3 in tumor infiltrating monocytes also is an attractive target for cancer therapy.

## Competing interests

The authors declare that they have no competing interests.

## Authors' contributions

WW, ZW, XM and CZ performed experiments; WW and JL designed research and wrote the paper; WW and ZW analyzed data. All authors read and approved the final Manuscript.

## Pre-publication history

The pre-publication history for this paper can be accessed here:

http://www.biomedcentral.com/1471-2407/11/506/prepub
